# Eighty per cent more patients in 10 years of UK molecular radiotherapy: Internal Dosimetry Users Group survey results from 2007 to 2017

**DOI:** 10.1097/MNM.0000000000001020

**Published:** 2019-05-02

**Authors:** Bruno Rojas, Daniel R. McGowan, Matthew J. Guy, Jill Tipping, Matthew Aldridge, Jonathan Gear

**Affiliations:** a Joint Department of Physics, Royal Marsden Hospital and Institute of Cancer Research, Surrey; b Radiation Physics and Protection, Oxford University Hospitals NHS Foundation Trust; c Department of Oncology, University of Oxford, Oxford; d Department of Medical Physics, University Hospital Southampton, Southampton; e Department of Nuclear Medicine, The Christie Hospital, Manchester; f Institute of Nuclear Medicine, University College London Hospital, London, UK

## Introduction

The Internal Dosimetry Users Group has surveyed UK molecular radiotherapy (MRT) activity for cancer treatment in the UK. Data are presented here spanning 10 years of MRT activity across 31 UK centres, one from Northern Ireland, one from Wales, two from Scotland and 27 from England.

Results were compiled from a survey of 2007 activity by the British Institute of Radiology Molecular Radiotherapy Working Party 1, subsequent matched surveys by Internal Dosimetry Users Group of data from 2011, 2012, 2013, 2014 and 2015 which have previously been presented 2,3 and new data from 2016 and 2017. The 2011 and 2012 surveys were carried out online using a dedicated webpage and subsequent surveys were distributed by email, all being publicized widely within the nuclear medicine and medical physics communities. Centres were asked a range of questions about MRT but presented here are data on the number of patients and treatments for each therapy type, activity regimens and trial participation. Treatments identified were Na^131^I radioiodine (RAI) for thyroid cancer, ^131^I-metaiodobenzylguanidine (^131^I-mIBG) and peptide receptor radionuclide therapy (PRRT) for neuroendocrine tumours (NETs), ^89^Sr-chloride, ^153^Sm-EDTMP and ^223^Ra-chloride (Xofigo; Bayer, Leverkusen, Germany) for bone metastases, MRT for haematological malignancies and selective internal radionuclide therapy (SIRT) for liver metastases.

## Results and discussion

**Fig. 1:**
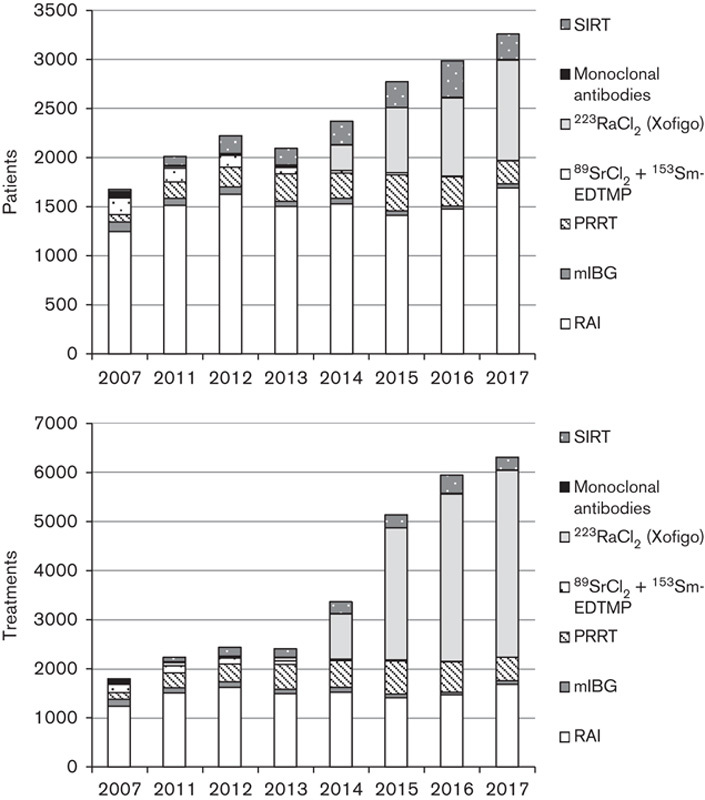
Total numbers of molecular radiotherapy patients treated (upper) and treatments given (lower) in each year of the survey for the 31 participating centres. mIBG, metaiodobenzylguanidine; PRRT, peptide receptor radionuclide therapy; RAI, radioiodine; SIRT, selective internal radionuclide therapy.

Between 2007 and 2017 we have seen an 82% increase in the number of patients treated using MRT and a 250% increase in the number of MRT treatments administered as shown in Fig. 1. All 31 responding centres provided MRT to adults and nine centres to paediatric patients as shown in Table 1. A total of 24 centres participated in 28 MRT trials which are summarized in Table 2 using the trial short name where available and the clinical trial IDs from the European Union (*https://www.clinicaltrialsregister.eu*), USA (*https://clinicaltrials.gov*) or UK (*http://www.isrctn.com*) registries in brackets.

**Table 1:**
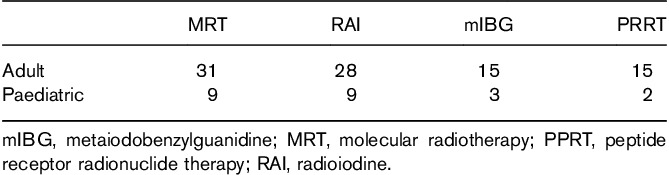
Number of centres from the 31 surveyed treating adult and paediatric patients with molecular radiotherapy, radioiodine for thyroid carcinoma and ^131^I-metaiodobenzylguanidine and peptide receptor radionuclide therapy for neuroendocrine tumours

**Table 2:**
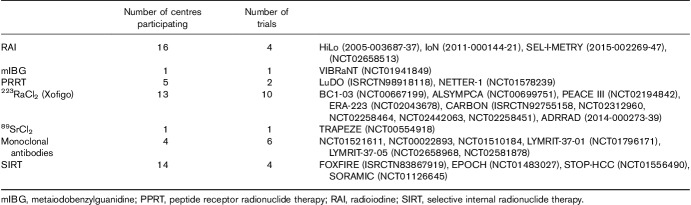
Trial participation in each molecular radiotherapy category within our group

**Fig. 2:**
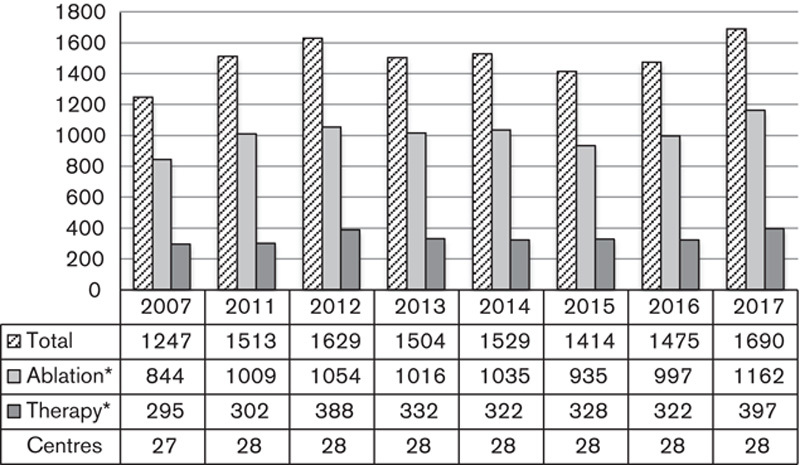
Numbers of radioiodine treatments for thyroid carcinoma with the number of centres administering. *Twenty-four centres were able to provide a breakdown of ablations and therapies.

Incidences of thyroid cancer have risen steadily over the past decade, increasing by 47% between 2007 and 2015 4. Survey data on RAI for thyroid cancer are summarized in Fig. 2 and initially reflected the steady rise in incidence. However, treatment numbers peaked in 2012 before declining up until 2015. Since then total treatments, made up of ‘Ablation’ (referring to the first RAI treatment after thyroidectomy intending to ablate residual thyroid) and ‘Therapy’ (referring to any subsequent RAI treatment) in a relatively consistent balance, have risen again reaching their maximum in 2017 where 35% more treatments were given than in 2007. The cause of the undulation is unclear although recruitment onto the HiLo trial 5 (recruiting from 2007 to 2010) may have acted to increase RAI referrals, while the IoN trial 6 (recruiting from 2012 to 2019) and most significantly changes in referral criteria in the British Thyroid Association guidelines 7 may have acted to temporarily reduce numbers.

**Fig. 3:**
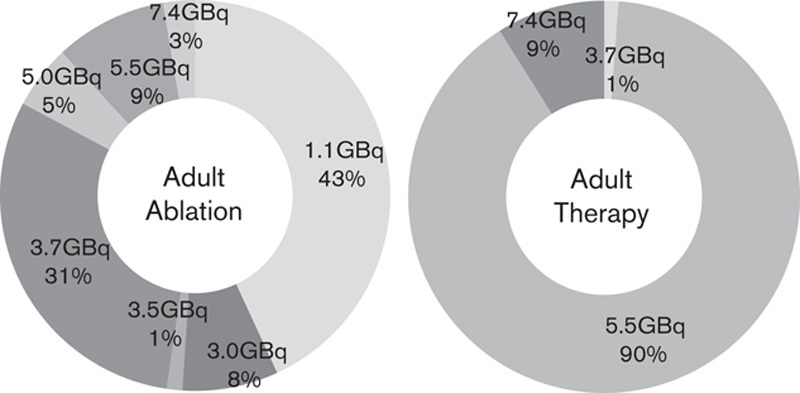
Distribution of activities given for adult ablation and adult therapy in 12 centres who were able to provide a breakdown of administrations in 2017.

The activity prescribed for RAI has shifted over the past 10 years, likely driven by the HiLo trial leading to the widespread use of 1.1 GBq, rather than 3.7 GBq, for low-risk ablation patients. In 2007, the modal activity given for an adult ablation was 3.0 or 3.7 GBq of ^131^I 1, in 2011 only four survey respondents indicated that they treated using 1.1 GBq, in 2017 this was true for all 28 centres providing RAI. Twelve centres were able to provide a numerical breakdown of the activities prescribed in 2017 to adult patients receiving ablation (411 patients total) and therapy (168 patients total) as shown in Fig. 3. The activities of ^131^I most frequently prescribed for adults were 1.1 GBq followed by 3.7 GBq for ablations, and 5.5 GBq for therapies. RAI is available to paediatrics in all nine centres treating paediatric patients with MRT. Data for paediatric treatments are insufficient to show the distribution of prescribed activities for ablation and therapy but centres indicated that they would prescribe 1.1–3.7 GBq for ablation and 3.7–5.5 GBq for therapy.

**Fig. 4:**
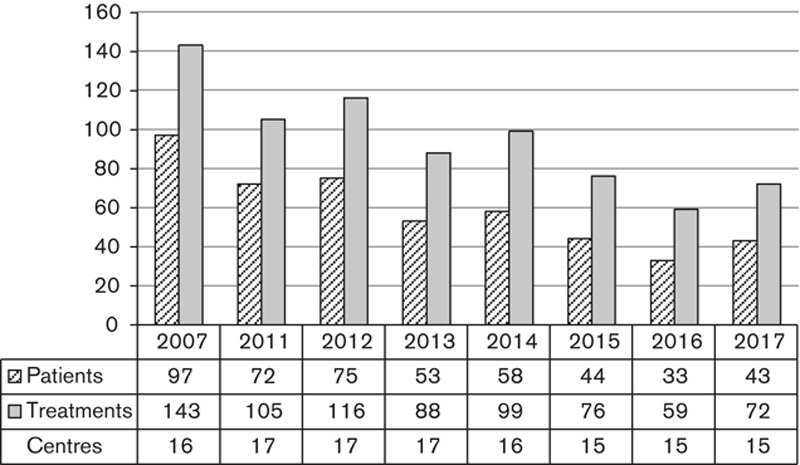
Total number of patients treated and treatments given using ^131^I-metaiodobenzylguanidine.

NET incidence has risen in the UK 8, as have the overall numbers of MRT treatments for NETs over the past 10 years in our surveyed centres. ^131^I-mIBG use has steadily declined as shown in Fig. 4, with the number of centres administering remaining relatively steady but generally giving fewer treatments each year, potentially as patients are instead referred for PRRT. Only one clinical trial was reported in our group using ^131^I-mIBG. However, two forthcoming ^131^I-mIBG trials, MiniVan (NCT02914405) and VERITAS (NCT03165292), are aimed at paediatric patients with neuroblastoma. ^131^I-mIBG remains an established treatment for neuroblastoma in this cohort, with two surveyed centres providing ^131^I-mIBG to paediatrics only.

**Fig. 5:**
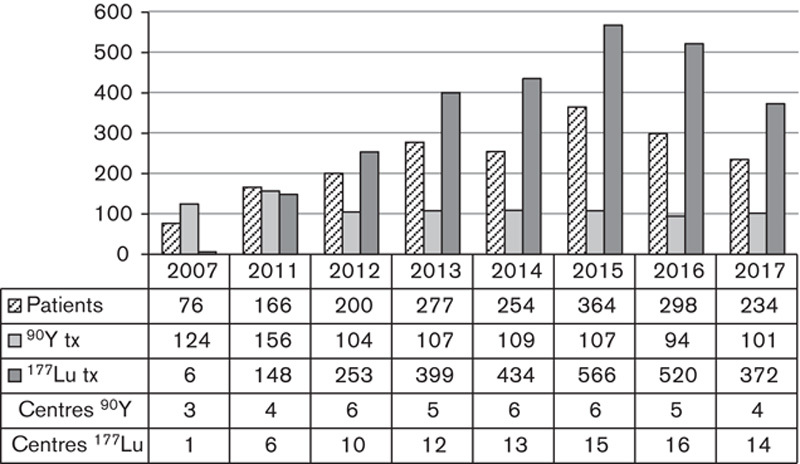
Total number of patients treated using peptide receptor radionuclide therapy, number of treatments (tx) given using ^90^Y-labelled and ^177^Lu-labelled radiopharmaceuticals and number of centres administering each.

In contrast with mIBG the use of PRRT for treatment of NETs had grown rapidly from 2007 to 2015 with a sharp decline since 2016 as is visible in Fig. 5. Once dominated by the use of peptides labelled in-house to ^90^Y, the wide-scale availability of prelabelled ^177^Lu-dotatate (Lutathera; Advanced Accelerator Application, Saint-Genis-Pouilly, France), has aided a rapid expansion in the use of PRRT. In 2007 only four centres provided PRRT, by 2016 there were a total of 17, of whom five provided ^90^Y-labelled peptides and 16 used Lutathera, four using both. From 2007 to 2015, the number of patients treated and treatments given grew almost five-fold. Lutathera was removed from the Cancer Drugs Fund (CDF, *https://www.england.nhs.uk/cancer/cdf/*) in November 2015 (*http://www.parliament.uk*), the impact of which is clearly visible in Fig. 5, with the number of Lutathera treatments given in our group falling by 2017 to two-thirds of the number given in 2015. Following positive results of the NETTIR-1 trial 9 Lutathera has recently had approval by the European Medicines Agency and The National Institute for Health and Care Excellence (NICE) for use in treating NETs 10 and a possible rise in treatments is predicted in the coming years.

**Fig. 6:**
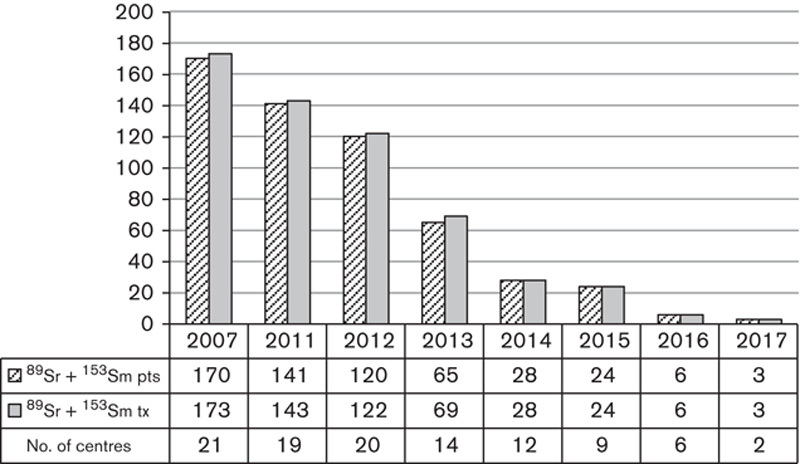
Number of patients (pts) treated, treatments given (tx) and number of centres administering ^89^Sr and ^153^Sm radiopharmaceuticals for bone metastases.

Palliation of bone pain with MRT was historically mainly limited to ^89^Sr-chloride and ^153^Sm-EDTMP treating at most 170 patients in the surveyed centres in 2007 as shown in Fig. 6. These treatments have steadily declined in popularity and are now rarely used. The small number administered in 2017 were to patients that were considered unsuitable for Xofigo therapy due to poor health or due to having soft tissue metastases.

**Fig. 7:**
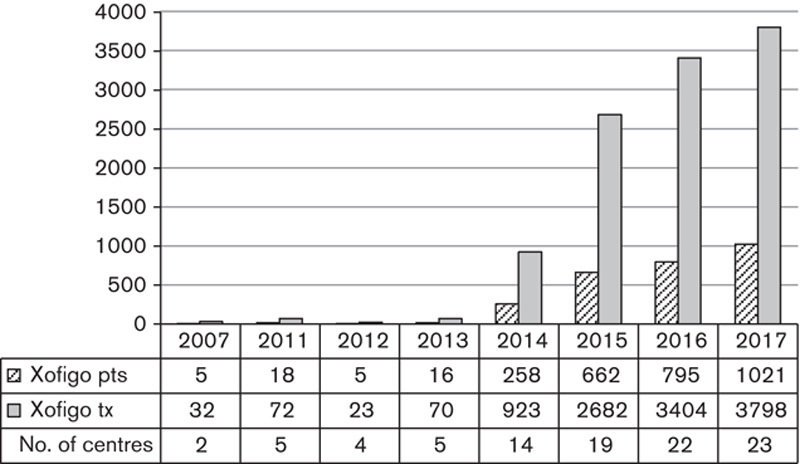
Number of patients (pts) treated, treatments given (tx) and number of centres administering Xofigo.

Following the positive findings of the ALSYMPCA trial 11, a period of availability on the CDF and NICE approval in 2016 12 the use of Xofigo has grown rapidly as shown in Fig. 7 and is now the most frequently administered form of MRT for cancer treatment. Xofigo has not only replaced ^89^Sr-chloride and ^153^Sm-EDTMP but is now used to treat roughly five times as many patients as were treated for bone metastases in 2007. Xofigo also features in the largest number of MRT trials across the survey cohort, including several phase II and III trials, as shown in Table 2. These trials are broadening the range of patients eligible for this treatment to include not just men with prostate cancer but also breast cancer patients.

It is worth noting that, as Xofigo treatments are given over the course of 6 months, our data on the number of patients are slightly inflated as centres will likely have counted patients starting their course in the previous year as well as those finishing in the next. In the latest survey centres were separately asked for the number of new patients treated in 2017, that is, patients who had their first Xofigo treatment in 2017. Providing that treatments are evenly spread across the year this question should yield a truer reflection of the number of patients. In 2017, our respondents treated 811 ‘new’ patients (compared with the 1021 ‘total’ patients), meaning that in the survey cohort the average Xofigo patient received 4.7 administrations out of the intended 6, most likely due to disease progression or complications rendering the patient unsuitable for further treatment.

MRT treatment for haematological malignancies has remained a small but highly varied area throughout the survey period. The largest number treated in any 1 year was 57 using ^90^Y-Zevalin in 2007, whereas other treatments, including three patients treated in 2017 with a ^227^Th-labelled agent, have been limited to small numbers from a relatively large number of trials (Table 2). Clinical trials of other monoclonal antibody therapies are now open across six UK centres and cover both paediatric and adult patient populations with haematological malignancies.

**Fig. 8:**
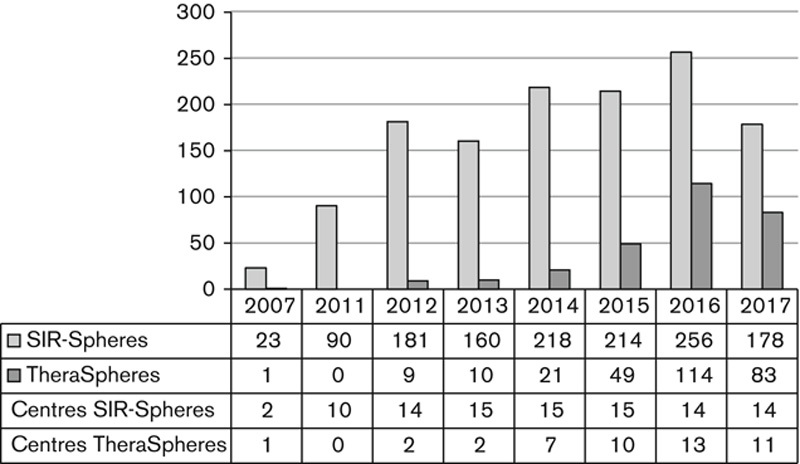
Total number of selective internal radionuclide therapy (SIRT) treatments per year, and centres treating using SIRTeX SIR-Spheres and BTG TheraSpheres.

The number of SIRT procedures increased rapidly over the survey period as shown in Fig. 8 following a surge in use of ^90^Y-SIR-Spheres (SIRTeX, Sydney, New South Wales, Australia), and later ^90^Y-TheraSpheres (Nordion, Ottawa, Ontario, Canada). Both agents were accompanied by trials in which surveyed centres participated (Table 2). Growth has not been steady however with SIRT being cut from the CDF in 2012, followed by a period of Commissioning through Evaluation between December 2013 and March 2017 13 during which funding was allocated to 10 centres for SIRT, nine of which are included in this survey. The concluding report of the Commissioning through Evaluation 13 and the FOXFIRE global trial results 14 reflected poorly on SIRT in its current application, chiefly to patients with advanced and heavily pretreated colorectal cancer. In 2017, the use of both SIRT agents decreased.

## Conclusion

MRT in 2017 was used to treat almost double the number of cancer patients as in 2007, with three and a half times as many treatments. Changes in funding have had a major short-term impact on the use of new therapeutics. However, the greatest changes have arguably been driven by a large number of multicentre phase II and III trials. In the treatment of NETs, the use of ^131^I-mIBG has declined, likely due to the rapid increase in the use of PRRT over recent years. PRRT growth has been backed by encouraging trial results, although not without significant upset due to the withdrawal of funding. A period of undulation in treatment numbers and a reduction in activity used to treat thyroid cancer with RAI may be driven by trials and updated guidelines. Unfavourable trial results appear to have had a detrimental impact on the use of SIRT which, despite unsteady funding over the survey period, had been rising. The most significant growth in any MRT category has also been accompanied by the largest number of clinical trials with Xofigo replacing and surpassing existing treatments for bone metastases in an expanding cohort of patients.

The extent of the success of new agents introduced over the past decade, accompanied by clinical trials providing a robust evidence base that most MRTs have historically lacked, are a model for future therapeutics. They give an indication of the potential population that can be reached when agents are backed up, and arguably publicized, by clinical trials, but also demonstrate the resource implications of new and expanding MRTs. Balancing both of these factors requires monitoring data, ideally through a complete and up to date database of UK MRT activity, and highlights the necessity to harness the treatment optimization potential of patient specific dosimetry.
